# Influenza and Common Colds

**Published:** 1890-05

**Authors:** 


### Influenza and Common Colds; the Causes, Character, and Treatment of each. By W. T. Ferrie, M. D. Percival & Co., King Street, Covent Garden, London, 1890. Price, 2 shillings.

This little book is mainly intended for the laity. The descriptions of the affections treated are terse and all that can be desired for the use intended. The treatment advised is a mixture of Homœopathy and allopathy. Still we may learn some useful hints from its pages. G. H. C.

